# The Effect of Corn Dextrin on the Rheological, Tribological, and Aroma Release Properties of a Reduced-Fat Model of Processed Cheese Spread

**DOI:** 10.3390/molecules27061864

**Published:** 2022-03-13

**Authors:** Christopher N. Schädle, Stephanie Bader-Mittermaier, Solange Sanahuja

**Affiliations:** 1 Aroma and Smell Research, Department of Chemistry and Pharmacy, Friedrich-Alexander University Erlangen-Nürnberg, Henkestraße 9, 91054 Erlangen, Germany; 2 Department of Food Process Development, Fraunhofer Institute for Process Engineering and Packaging IVV, Giggenhauser Str. 35, 85354 Freising, Germany; stephanie.mittermaier@ivv.fraunhofer.de; 3 School of Agricultural, Forest and Food Sciences (HAFL), Bern University of Applied Sciences, Länggasse 85, 3052 Zollikofen, Switzerland; solange.sanahuja@bfh.ch

**Keywords:** tribology, rheology, lubrication, viscosity, proton-transfer-reaction mass spectrometry (PTR-MS), dietary fiber, texture, structure, flavor release, cheese aroma

## Abstract

Low-calorie and low-fat foods have been introduced to the market to fight the increasing incidence of overweightness and obesity. New approaches and high-quality fat replacers may overcome the poor organoleptic properties of such products. A model of processed cheese spread (PCS) was produced as a full-fat version and with three levels of fat reduction (30%, 50%, and 70%). Fat was replaced by water or by corn dextrin (CD), a dietary fiber. Additionally, in the 50% reduced-fat spreads, fat was replaced by various ratios of CD and lactose (100:0, 75:25, 50:50, 25:75, and 0:100). The effect of each formulation was determined by measuring the textural (firmness, stickiness, and spreadability), rheological (flow behavior and oscillating rheology), tribological, and microstructural (cryo-SEM) properties of the samples, as well as the dynamic aroma release of six aroma compounds typically found in cheese. Winter’s critical gel theory was a good approach to characterizing PCS with less instrumental effort and costs: the gel strength and interaction factors correlated very well with the spreadability and lubrication properties of the spreads. CD and fat exhibited similar interaction capacities with the aroma compounds, resulting in a similar release pattern. Overall, the properties of the sample with 50% fat replaced by CD were most similar to those of the full-fat sample. Thus, CD is a promising fat replacer in PCS and, most likely, in other dairy-based emulsions.

## 1. Introduction

The consumption of high-energy foods and beverages, combined with a sedentary lifestyle is the main cause of overweightness and obesity worldwide, which are associated with health concerns, such as high blood pressure, type 2 diabetes mellitus, cancer, and cardiovascular diseases, as well as, subsequently, high healthcare costs [1,2,3]. Foods with lower calories can help to prevent such diet-related health issues. Processed cheese products are consumed all over the world and the market value was estimated to reach USD 24,000,000,000 by 2029 worldwide [4], and thus, a high potential for the reduction of fat can be anticipated. Processed cheese products are stable oil-in-water emulsions, supported by a gel network of hydrated and emulsified casein proteins, whereby the fat content is mainly responsible for its texture, color, and taste [5,6,7,8,9]. The main difference between processed cheese, as slices or blocks, and processed cheese spread (PCS) is the moisture content [10]. The basic raw material for conventional processed cheese is natural cheese, which is subsequently mixed with other dairy and non-dairy ingredients, such as butter, cream, (skim) milk powder, water, and emulsifying salts [11]. Natural cheeses mainly consist of milk fat, protein, such as caseins, and moisture. Generally, fat provides the major source of calories in processed cheese [12]. In this study, we used a model of PCS where we replaced the natural cheese base by adding its main components as single ingredients. Therefore, rennet casein, water, and a vegetable milk-fat-alternative were used to obtain the model PCS, which could be easily reduced in fat. The effect of dry matter, protein, and fat content on the textural, rheological, and structural properties of PCS has been studied by several researchers [13,14,15]. Previous findings suggest that the reduction of fat in cheese causes structural, functional, and sensory deficiencies [16,17] and high-quality fat replacers are necessary to develop appealing and tasty products. Up until now, no ingredient has been able to replace all the fat properties in processed cheese products. According to Ferrão et al. [12], starch is one of the main fat replacers used in the foods industry. However, when starch is used, the maintenance of the sensory properties of the reduced-fat products is often associated with an insignificant calorie reduction. Corn dextrin (CD) is a fat replacer produced by the partial hydrolysis of corn starch. Furthermore, it is classified as a water-soluble dietary fiber with a reduced calorie content, compared to starch. Hence, besides its fat replacer properties, it meets consumers’ growing demand for fiber-containing foods [18]. It is also known for its low glycemic and insulinemic responses, digestive comfort, and prebiotic effects [19]. CD has been used as a promising fat replacer in emulsion-based dairy products [20,21,22], but not yet in PCS.

Dynamic oscillatory rheological measurements are used to determine the viscoelastic behavior, which, in cheese, is mostly dictated by the properties of the principal component forming the continuous network—the protein network. The bonds among proteins are primarily responsible for the elastic properties of cheeses. Other ingredients can modify the properties of the network in different ways [23]. Frequency tests can be used to determine gel properties and classify them into entangled networks (of biopolymers), chemical (cross-linked) gels, or physical (noncovalent linkage) gels. Soft gels, such as entangled networks, show viscoelastic moduli with a strong dependence on frequency, and may have fluid-like properties at low frequencies and solid-like properties at higher frequencies and, thus, a G′–G″ crossover. Cross-linked gels possess a covalent network and little frequency dependence, while physical gels are intermediate between strong and weak gels. The latter have some frequency dependence, but no G′–G″ crossover [24,25,26]. Texture analyses and rheological measurements are often used to describe the characteristics of food products, but additional methods are necessary to capture the further relevant sensory attributes of a product that humans experience during oral processing [27]. The oral perception at the front of the oral cavity is initially dominated by the bulk properties, such as deformation and breakdown mechanics for solids (instrumental texture analysis) or overall flow for fluids (rheology), due to a rather large gap between the tongue and the palate [28]. During oral processing and swallowing, food is mixed with saliva, and squeezed and sheared between the surfaces of the tongue and palate. The perception is dominated by a thin film rheological behavior, which can be determined by tribological measurements. Tribology is the study of the friction and lubrication of interacting surfaces in relative motion with, or without, a lubricant in-between [29]. It records the coefficient of friction (COF) versus the sliding speed, resulting in the so-called Stribeck curve. Schädle et al. [21] have shown that tribological measurements of reduced-fat emulsions are able to describe lubrication properties and predict sensory evaluations of attributes, such as stickiness. The Stribeck curve is divided into a static region, followed by a kinetic region with an increasing sliding speed. The kinetic region, in turn, is separated into the boundary, the mixed friction, and the (elasto)-hydrodynamic regime [30].

In addition to the physical and technological properties of foods, the effect of fat reduction on aroma release also plays a major role. The six aroma compounds in this study (butane-2,3-dione (diacetyl), butanoic acid (butyric acid), ethyl butanoate (ethyl butyrate), 3-methylbutanoic acid (isovaleric acid), heptan-2-one, and nonan-2-one) represent typical aroma compounds in various cheeses and other milk products [31,32,33,34,35] and possess different chemical structures and properties, such as solubility and hydrophobicity. Schädle et al. [22] demonstrated different influence factors on the aroma release of these six aroma compounds from full-fat and reduced-fat model emulsions. The fat content had a larger impact than the viscosity of the emulsions. Furthermore, hydrophobic aroma compounds were more affected by fat reduction. The use of the fat replacers, CD and microparticulated whey protein, provided reduced-fat emulsions with similar aroma release patterns to the full-fat emulsion. The properties of the aroma compounds and the food matrix determine the release rate of the compounds from the food matrix in the air [36]. Additional factors are involved in the in vivo aroma release, such as the influence of saliva (quantity and composition) and the oral processing (mastication, mixture with saliva, and bolus formation).

In this study, a full-fat model of PCS was compared to reduced-fat variations. Fat was reduced in three reduction levels (30%, 50%, and 70%) and was replaced either by water or by CD. In addition, the samples with a 50% fat reduction were produced with various ratios of CD and lactose (100:0, 75:25, 50:50, 25:75, and 0:100) to replace the fat. Lactose was used as an inert filler [37] to maintain the dry matter content of the model of PCS and to validate the fat replacer properties. The effect of fat reduction and the use of CD as a fat replacer was determined by analyzing the textural (firmness, stickiness, and spreadability), rheological (flow behavior and oscillating rheology), and tribological properties. Furthermore, the microstructure (cryo-SEM) of the samples, as well as the dynamic aroma release of the six aroma compounds prevalent in cheese, were determined to investigate the impact of the fat replacement in detail. The results aim to bring about new approaches to reduce fat in PCS and its related products with organoleptic and technological properties similar to the full-fat product.

## 2. Results and Discussion

### 2.1. Composition

The full-fat and all reduced-fat PCS samples with CD and lactose had a dry matter content between 36 and 37 g/100 g, with no significant differences among them. As expected, the dry matter content of the reduced-fat PCS that contained solely water for the replacement of fat was lower, with about 24 g/100 g for the Fatred 70, about 27 g/100 g for the Fatred 50, and about 30 g/100 g for the Fatred 30 samples, respectively. The absolute fat content was 20.9 ± 0.5 g/100 g in the full-fat sample, and was approximately halved in the samples Fatred 50 and Y CD 50, reduced by about 30% in the samples Fatred 30 and 100 CD 30, and reduced by about 70% in the samples Fatred 70 and 100 CD 70. The absolute protein content was the same for all samples, at about 8.9 g/100 g, because all protein-containing ingredients were the same in all formulations. The full-fat sample had the highest energy density of about 942 kJ/100 g (228 kcal/100 g), which was reduced by about 30% for the 100 CD 50 sample to 656 kJ/100 g (158 kcal/100 g). A table with all the values for fat, protein, and dry matter content, as well as the energy densities of all formulations, can be found in the Appendix A (Table A1). The pH value was at pH 6.0 for all samples.

### 2.2. Texture Analysis

**Figure 1:**
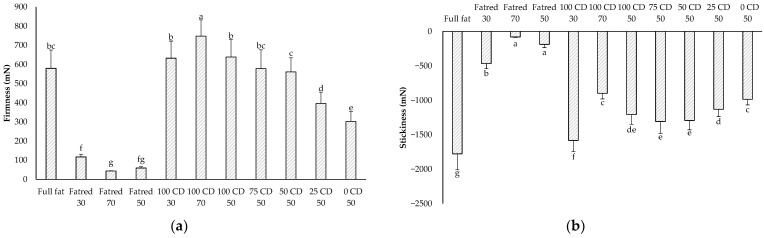
Firmness (**a**) and stickiness (**b**) of the PCS samples measured with the Texture Analyzer (CD = corn dextrin). The data are expressed as mean ± standard deviation (*n* > 24). Bars with different letters indicate significant differences between samples (*p* ≤ 0.05) following one-way ANOVA (Tukey).

Firmness and stickiness are important attributes with an impact on the mouthfeel [38] and the processability of food products. The firmness of the full-fat PCS sample was about 580 mN (Figure 1a) and was reduced significantly by replacing parts of the fat by water in the Fatred X samples. This is most probably due to a dilution of the protein network by the higher water content, as well as the removal of the fat itself, which is partly present as solid fat at this temperature. The lower the fat content, the lower the firmness of the samples. All other reduced-fat samples had the same dry matter content as the full-fat sample. However, they differed partly in their firmness, which might be attributed to the impact of other ingredients, most likely the lactose content. In the 100 CD 70 sample, 70% of the fat was replaced by CD and had a significantly higher firmness than all other samples. In the samples with a 30% and 50% fat replacement, the samples with 100% or 75% CD (100 CD 30, 100 CD 50, and 75 CD 50) had the same firmness as the full-fat sample. Replacing the fat by a lower CD concentration and, thus, a higher lactose concentration yields a significantly lower firmness. Therefore, solely balancing the dry matter content of reduced-fat products is not sufficient to gain similar properties to the full-fat product. However, CD showed fat-like properties in solidifying the PCS samples at the 30% and 50% fat reduction levels.

The stickiness of the PCS samples is shown in Figure 1b. The higher the absolute value of the stickiness, the stickier and more cohesive the samples [39]. The full-fat sample exhibited a significantly higher stickiness, and the Fatred X samples were the least sticky, indicating that fat intensifies stickiness, while water reduces it. However, Ningtyas et al. [40] found a higher firmness, spreadability, and adhesiveness for low-fat, compared to high-fat, cream cheese, although the moisture content was also higher in the low-fat sample. In contrast to our study, they used different protein concentrations, and their higher values might be explained by the higher protein content in the low-fat sample. The addition of CD or lactose in our investigation did not lead to a similar stickiness compared to the full-fat sample. However, the Y CD 50 samples exhibited higher stickiness values compared to the Fatred 50 sample, and the addition of CD or lactose led to a higher stickiness, probably due to a higher dry matter content and, thus, a higher cohesiveness in these samples as a result of higher molecular interactions. In addition, the samples with higher CD concentrations were again closer to the full-fat sample than the samples with higher lactose concentrations.

### 2.3. Spreadability

**Figure 2:**
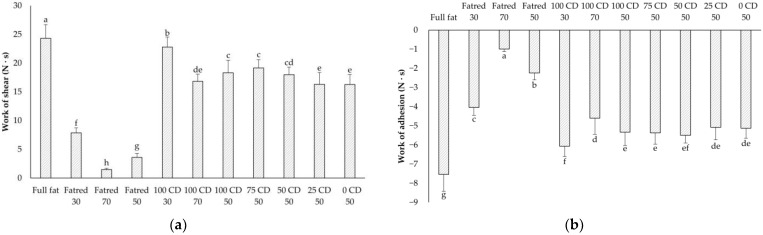
Work of shear (**a**) and work of adhesion (**b**) of the PCS samples measured with the Spreadability Rig sample probe on the Texture Analyzer. The data are expressed as mean ± standard deviation (*n* > 24). Bars with different letters indicate significant differences between samples (*p* ≤ 0.05) following one-way ANOVA (Tukey).

The spreadability of the PCS samples was analyzed with the Spreadability Rig. The work of shear, and the work of adhesion of the samples were determined (Figure 2a,b).

The work of shear is intended to describe the physical work required to spread the model PCS, e.g., with a knife on bread. It was expected that samples with a higher firmness simultaneously would need a higher work of shear to be spread, and indeed, the Fatred X samples behaved in this manner. The lower the fat content in these samples was, the lower the firmness and the work of shear. Interestingly, the samples with CD behaved slightly different. While the firmness increased with a lower fat and higher CD content in the 100 CD X samples, the work of shear behaved exactly inversely. CD appeared to have a lower impact on spreadability than on the firmness of the model PCS, probably due to a better lubrication, as described in Section 2.7. Overall, the higher the fat content in the Fatred X or the 100 CD X samples, the higher the work required to spread the samples. The samples with CD and lactose were significantly easier to spread compared to the full-fat model PCS. The work of adhesion is a measure of the adhesion of the PCS to surfaces, for example a knife. Here, a similar behavior of the PCS was determined for the stickiness of the samples.

### 2.4. Flow Behavior

**Figure 3:**
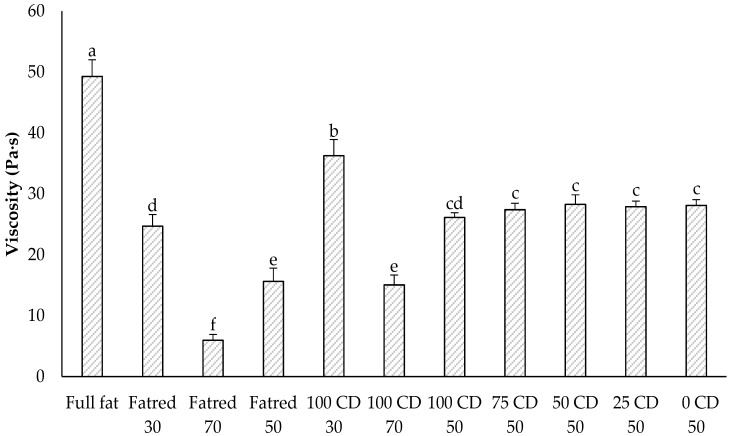
Viscosity of the PCS samples determined with an RVA. The data are expressed as mean ± standard deviation (*n* > 12). Bars with different letters indicate significant differences between samples (*p* ≤ 0.05) following one-way ANOVA (Tukey).

Rheological properties, such as viscosity, are often affected by the fat content in foods [41]. Therefore, the apparent viscosity was determined at a given shear rate as an indicator of flow behavior. Generally, the full-fat sample exhibited the highest viscosity of all model PCS samples (Figure 3). All reduced-fat samples showed significantly lower viscosities, which is attributed to the reduced solid fat contents. Furthermore, no significant difference was determined between the impact on viscosity of either adding a fat replacer or adding lactose.

### 2.5. Dynamic Oscillatory Rheology—Frequency Sweep

The samples 100 CD 70 and 100 CD 50 both exhibited a dominant storage modulus over the entire frequency range, and the sample 75 CD 50 demonstrated similar values for the storage and loss moduli at lower frequencies, as well as a dominant storage modulus at higher frequencies. The full-fat, Fatred 30, Fatred 50, 100 CD 30, 50 CD 50, 25 CD 50, and 0 CD 50 samples showed a dominant loss modulus at low frequencies and changed their properties at higher frequencies towards a more elastic behavior with a dominant storage modulus. The lower the CD concentration and the higher the water content, the higher the frequency at which the crossover took place, resulting in the following order from low to high frequencies for the crossover point: 50 CD 50, 100 CD 30, 25 CD 50, 0 CD 50, full-fat, Fatred 30, and Fatred 50. The sample Fatred 70 was the only sample with a dominant loss modulus over the whole frequency range, indicating a more fluid than gel-like behavior. Rennet casein probably formed a cross-linked protein gel in all samples, except for the Fatred 70 sample, where the high amount of water diluted the proteins, resulting in a very loose network. Adding CD to the samples led to a more entangled network with physical, noncovalent linkages at very high CD concentrations.

The value A_F_ is a measure of gel strength. The highest gel strength was found for the full-fat sample, followed by the three 100 CD X samples (Table 1). The gel strength decreased with decreasing CD concentrations and was lowest for the Fatred X samples. Although the protein concentration was identical in all samples, the Fatred X samples differed in their protein-to-water ratio. Lee et al. [15] described a change in the model processed cheese rheological properties from a concentrated liquid to a weak gel, and an increased firmness with an increasing protein-to-water concentration, which is in accordance with our results. The interaction factor z was highest for the 100 CD 70 sample; thus, this was the sample with the highest CD concentration, indicating a high interaction of CD molecules with other ingredients or with themselves. The factor decreased with decreasing CD concentrations and was lowest for the Fatred X samples. Černíková et al. [42] found an increasing gel strength and interaction factor with an increasing dry matter content and with a decreasing fat content. While the relationship between gel strength, as well as the interaction factor with dry matter content, could be found for our samples, the relationship with the fat content was not detectable. This could be due to an increasing water content in the Fatred X samples, or an increasing CD concentration in the 100 CD X samples with a decreasing fat content. The samples 100 CD 70 and 100 CD 50, with the highest interaction factor, also showed a dominant storage modulus without a G′–G″ crossover over the entire frequency range, indicating an entangled network structure. In contrast, the sample Fatred 70 with the lowest interaction factor showed a continuous dominant loss modulus over the entire frequency range.

**Figure 4:**
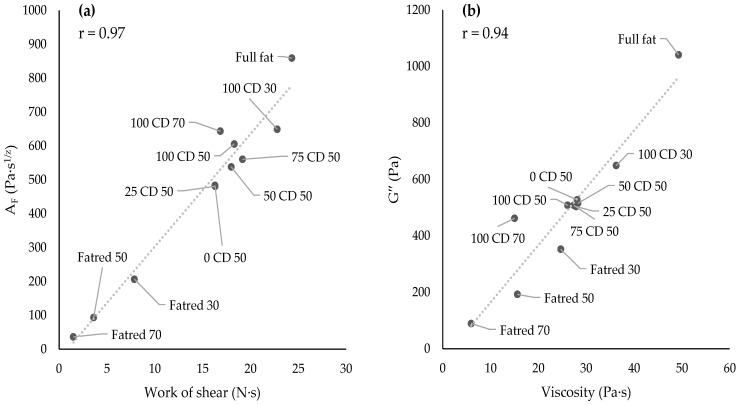
Correlation between work of shear (spreadability) and gel strength A_F_ (**a**) as well as between loss modulus and viscosity (**b**) (r = correlation coefficient).

Additionally, the G′, G″, G*, and tan δ values were determined at a reference frequency of 1 Hz [11,43] (Table 1). The order of the samples, according to their loss tangent values, corresponded to the order of crossover frequency and the values of the interaction values described above. The higher the interaction factor, the lower the frequency at the crossover point and the lower the loss tangent; thus, this led to a decreasing ratio of loss to the storage modulus. This is also consistent with the behavior of the samples with increasing CD concentrations. A higher CD concentration led to a higher interaction factor, a higher gel strength, and a lower loss tangent value. The order of the samples, according to their complex modulus G*, corresponded to the order of their gel strength A_F_ and of their storage modulus G′, while there were no significant differences in their loss moduli. The loss modulus describes the viscous properties of the samples, which correlated very well (r = 0.94) with the viscosity (cf. Section 2.4) (Figure 4b). CD and lactose appeared to have the same effect on viscosity, but differed in their effect on the elastic properties of the PCS samples. Gabriele et al. [44] and Piska and Štětina [43] also found a corresponding increase in the values of G′, G*, and A_F_ and related it to the rising gel strength of their semi-solid model samples, ranging from yoghurt, dough, and jams to processed cheese. We found a very high correlation (r = 0.97) between gel strength A_F_ and the work of shear (Figure 4a), indicating that a higher force is required to spread PCS samples with higher gel strength. Strohmaier et al. [45] reported a good correlation of oscillatory rheological measurements and spreadability in human sensory tests for processed cheese.

### 2.6. Dynamic Oscillatory Rheology—Temperature Sweep

**Figure 5:**
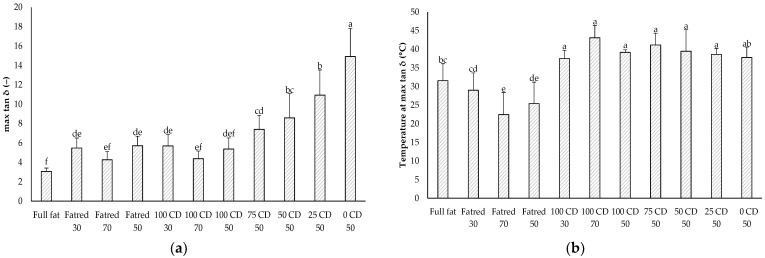
Maximum loss tangent (tan δ) (**a**) and temperature at the maximum loss tangent (**b**) of the PCS samples determined with a temperature sweep. The data are expressed as mean ± standard deviation (*n* > 9). Bars with different letters indicate significant differences between samples (*p* ≤ 0.05) following a one-way ANOVA (Tukey).

In addition to the frequency sweep, a temperature sweep was performed to determine the temperature-dependent viscoelastic behavior of the PCS samples. The maximum loss tangent is a measure of flowability, i.e., the ability to flow at a certain temperature [7,46,47]. The higher the value, the higher the degree of flowability. No significant differences were observed between all Fatred X and 100 CD X samples (Figure 5a). However, the maximum loss tangent increased with decreasing CD and increasing lactose concentrations. The addition of CD reduced the extent of flow at heating, while flowability increased with an increasing lactose content. A reduced flowability with increasing CD concentrations was also found by Schädle et al. [20] for heated processed cheese slides. Interestingly, the temperature at which the maximum loss tangent occurred was not significantly different between the reduced-fat samples with CD or lactose (Figure 5b). Hence, with lactose, instead of CD, the PCS flowed to a greater degree at the same temperature. However, with decreasing fat content and, thus, with an increasing water content, the temperature was reduced in the Fatred X samples. The addition of CD to the reduced-fat samples in the 100 CD X samples did not affect the flowability, but the samples required higher temperatures to achieve the same flowability as the samples without CD (Fatred X). Furthermore, the full-fat sample showed a significantly lower temperature, which coincides with the slip melting point of the used vegetable fat of about 30 °C, according to its specification.

**Figure 6:**
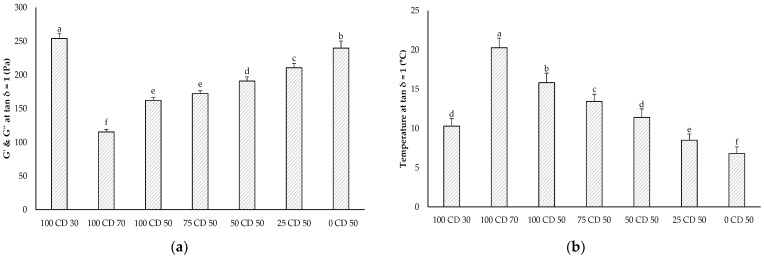
G′ and G″ at gel–sol transition temperature (**a**) and gel–sol transition temperature (**b**) of the PCS samples measured with a temperature sweep. The data are expressed as mean ± standard deviation (*n* > 9). Bars with different letters indicate significant differences between samples (*p* ≤ 0.05) following a one-way ANOVA (Tukey).

The gel–sol transition occurs at tan δ = 1, that is, when the curves of G′ and G″ intersect. The temperature at the gel–sol transition is a measure of the structural weakening during heating and an indicator of the meltability of cheese [48]. The storage and loss modulus at the gel–sol transition (G′ = G″), and the corresponding temperature of the samples containing CD, are shown in Figure 6. The full-fat and Fatred X samples did not show any intersection of the storage and loss moduli in the temperature sweep and, thus, are not displayed in the figure. The gel–sol transition temperature increased with increasing CD concentrations; thus, the PCS samples started to melt at higher temperature. Conversely, the storage and loss moduli at the gel–sol transition were higher for lower CD concentrations. Because the gel–sol transition temperature was lower for lower CD concentrations, the solid-fat content in these samples was higher and, thus, their storage and loss moduli were higher.

### 2.7. Tribological Properties

**Figure 7:**
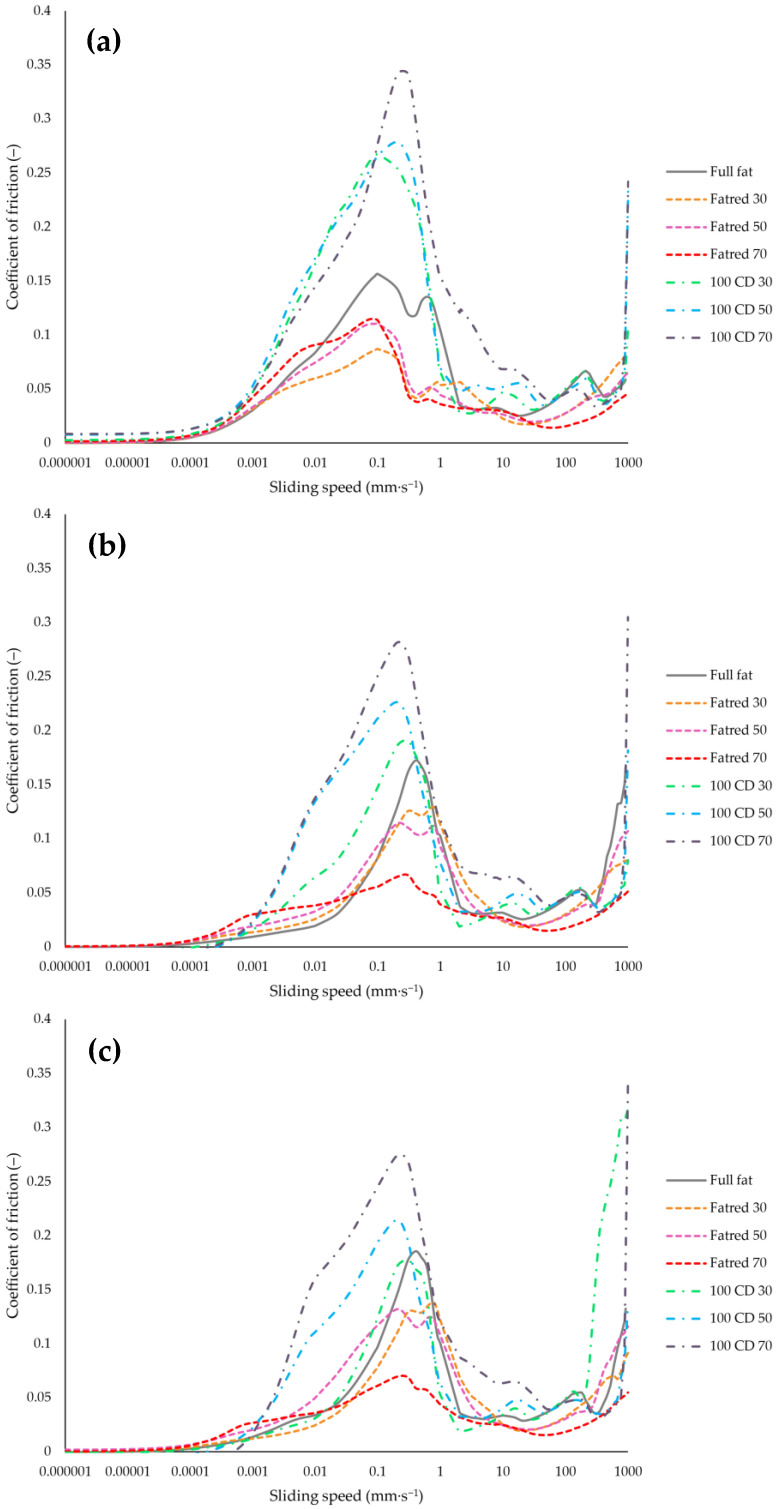
Stribeck curve 1 (**a**), curve 2 (**b**), and curve 3 (**c**): coefficient of friction versus sliding speed of PCS samples with different fat contents. The curves are mean values of all four measurements per each batch.

**Figure 8:**
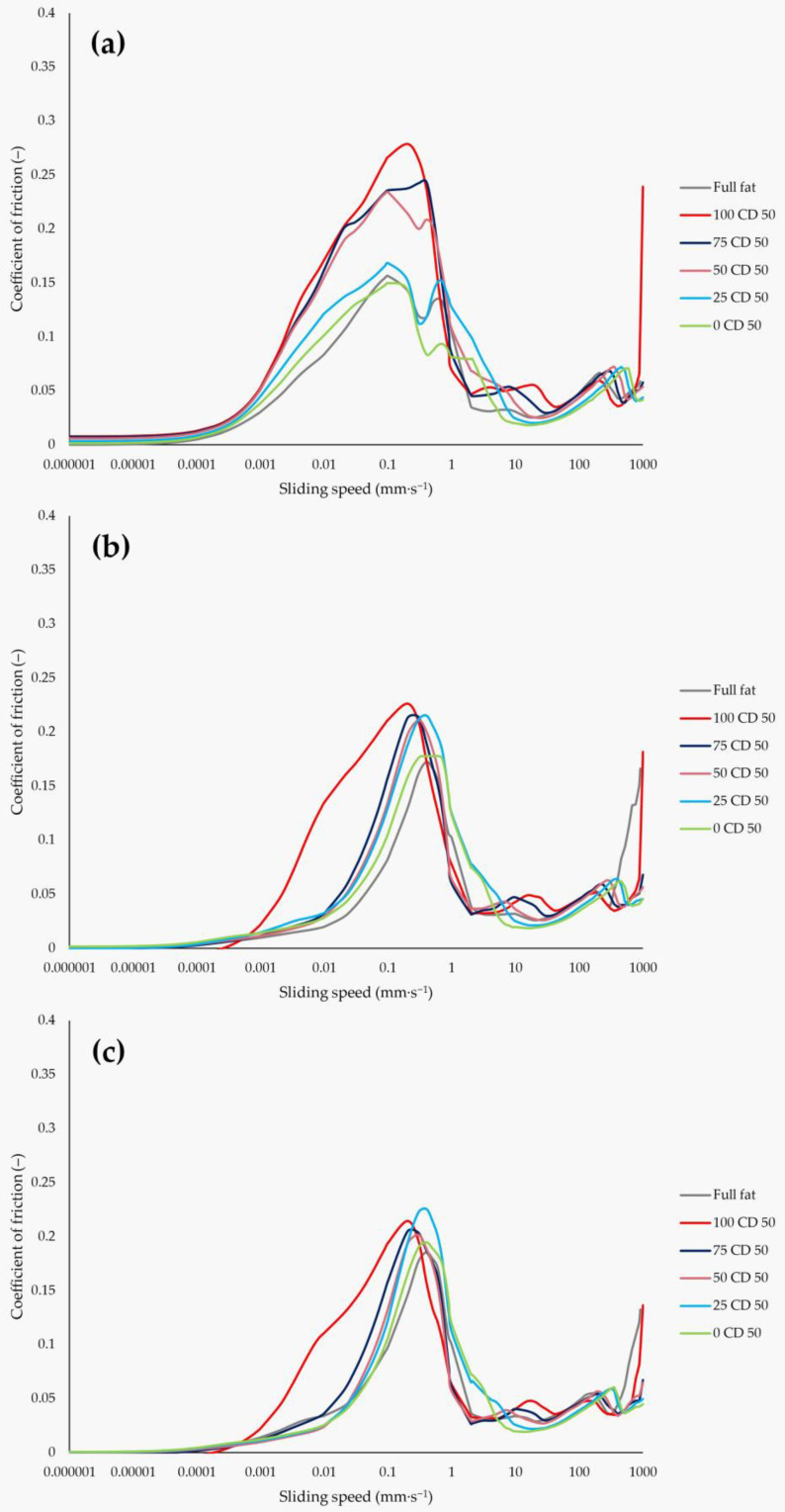
Stribeck curve 1 (**a**), curve 2 (**b**), and curve 3 (**c**): coefficient of friction versus sliding speed of PCS samples with different CD concentrations. The curves are mean values of all four measurements per each batch.

The tribological properties of the PCS samples are described by the COF versus the sliding speed, as shown in the Stribeck curves in Figure 7 and Figure 8. Tribological measurements are intended to describe the influence of food on the friction between the tongue and palate in a relative motion. Figure 7 shows the Stribeck curves of the full-fat, the Fatred X, and the 100 CD X samples. Interestingly, in the boundary regime, the samples with water replacing fat (Fatred X) showed a lower COF at the maximum compared to all other samples. One would assume a better lubrication of samples with a higher fat content. However, the full-fat sample with the highest fat content (20 g/100 g) showed a higher COF at the maximum than the Fatred X samples. Nevertheless, a very high correlation (r = 0.90 to 0.98) was observed between the interaction factor and the maximum COF of all three curves (Figure A2). The lubrication properties of the PCS samples in the boundary regime were governed by the interaction of structure units in the network, as higher degrees of interaction resulted in a higher COF. Within the 100 CD X samples, in turn, a correlation of a higher fat content and a lower CD content, with better lubricity, was observed. The differences among all samples became smaller in the sliding speed range between 2 and 200 mm∙s^−1^. Researchers suggested this sliding speed range to describe mouth-like conditions during food consumption [49,50,51], indicating that our samples might be rated similar in a sensory test.

Figure 8 shows the Stribeck curves 1, 2, and 3 of the Y CD 50 samples with different CD and lactose concentrations, as well as the full-fat sample as a reference. In the 0 CD 50 sample, 50% of the fat was replaced by lactose and no CD was added. This sample showed a COF at the maximum, which is the most similar to the full-fat sample. With increasing CD concentrations in the samples, the maximum COF increased with the highest COF for the 100 CD 50 sample. The differences between the samples also became smaller from curve 1 to curve 2 and 3. Furthermore, the curves also changed the least in the speed range of 2 to 200 mm∙s^−1^, such as for the samples in Figure 7. Interestingly, all samples in both Figure 7 and Figure 8 showed a local minimum between 0.3 and 0.5 mm∙s^−1^ in curve 1, except for the 100 CD X samples. The local minimum might indicate the coalescence of the fat droplets and the breakdown of the emulsion [52]. Regardless of the formulation, the coalesced fat formed a fat film on the surfaces of the tribo-pair. The fat film remained at the surfaces, which could be observed in a reduced COF and a smaller peak width in the boundary regime of curve 2 and 3 in both Figure 7 and Figure 8. The exceptions to this were the 100 CD X samples, which showed no local minima in the range of 0.3 to 0.5 mm∙s^−1^ and whose peak width was also barely reduced in curve 2 and 3, compared to curve 1 (Figure 7). Furthermore, the higher the CD concentrations in these samples, the smaller the variation of the COF between curve 1, 2, and 3 of each sample. The 100 CD 70 sample with the highest CD concentration of all samples showed the highest COF and the lowest change between the three curves. This could be due to a higher stabilization of the PCS emulsion with higher CD concentrations and, thus, a lower tendency of an emulsion breakdown during shearing. Thus, replacing fat with increasing CD concentrations produced PCS with an increased resistance against fat coalescence and, hence, against emulsion breakdown. Similar local minima were also found in the Stribeck curves of model emulsions [22], and could also be seen for mayonnaise samples [21] with different fat contents and fat replacers. However, for the mayonnaise sample, this was not detected until curve 2 or 3, indicating a stable emulsion. Only parts of the fat coalesced at the early stages, leading to a lower friction in the boundary regimes in the following curves 2 and 3, but the emulsion breakdown and enhanced fat coalescence occurred only after prolonged stress during the tribological measurement [21]. It appears that only curve 1 describes the properties of the PCS emulsion in its original state, while, in the case of curve 2 and 3, most of the PCS emulsions were already broken. Apparently, only curve 1 describes the samples as they are processed in the mouth. This should be investigated in further studies.

**Figure 9:**
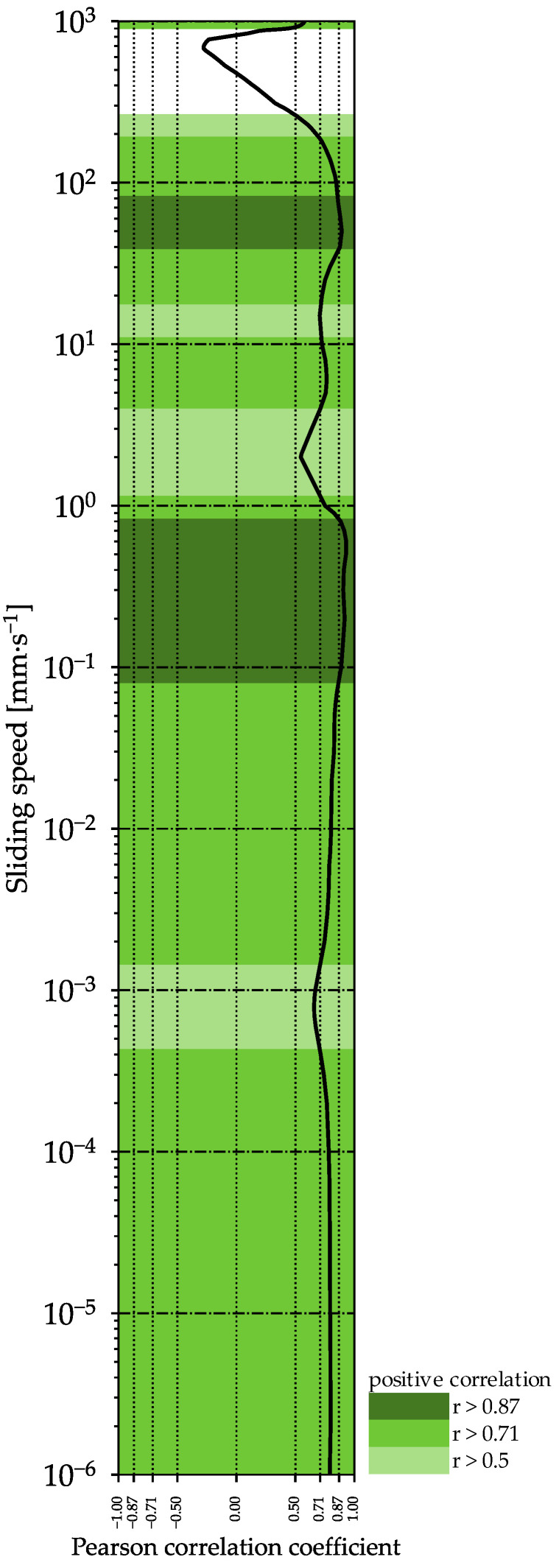
Pearson correlation coefficient for the interaction factor z and the coefficient of friction (COF) over the entire sliding speed range for curve 1 of the tribological measurements.

**Figure A1:**
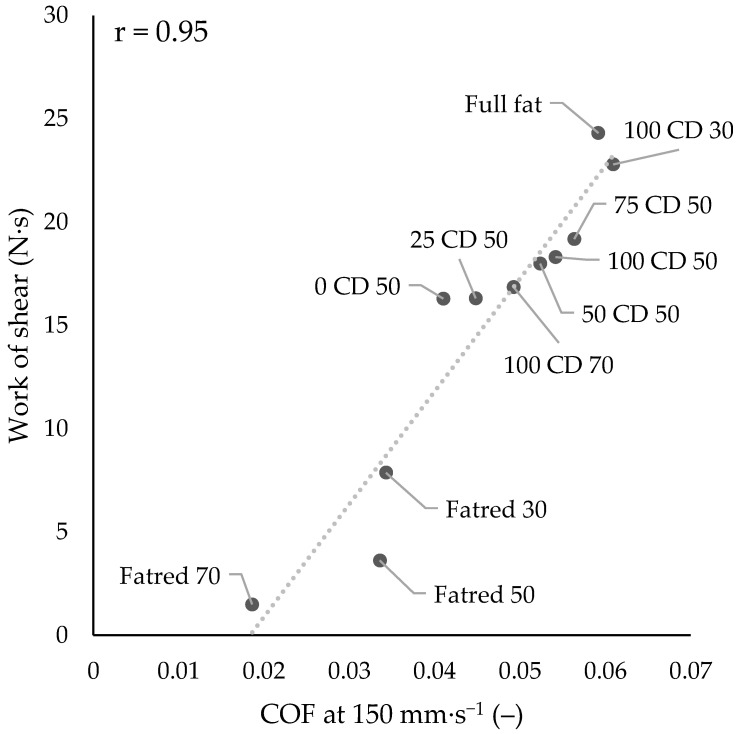
Correlation between work of shear and the COF at a sliding speed of 150 mm∙s^−1^ (r = correlation coefficient).

**Figure A2:**
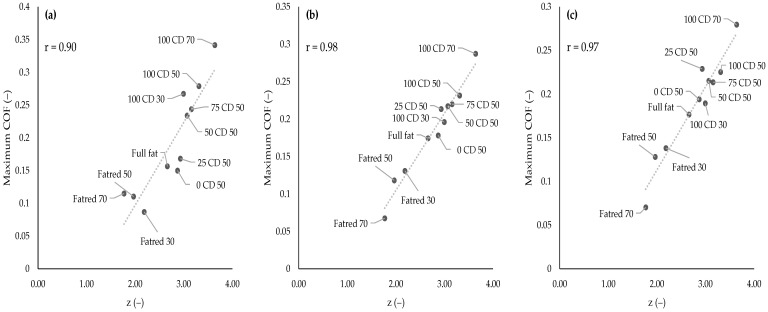
Correlations between the interaction factor z and the maximum COF of curve 1 (**a**), curve 2 (**b**), and curve 3 (**c**) (r = correlation coefficient).

Figure 9 shows the Pearson correlation coefficient between the COF of the samples, and the interaction factor z, at individual sliding speeds over the entire speed range of curve 1. The coefficient demonstrates a very high correlation between the two results over almost the entire speed range. The higher the interaction factor, the higher the COF of the sample. Thus, the interaction factor determined by oscillating rheological measurements may be used for the prediction of lubrication properties. Furthermore, a very high correlation (r = 0.95) was found between the work of the spread and the COF at a sliding speed of 150 mm∙s^−1^ (Figure A1), indicating a possible option to predict spreadability by tribological measurements.

### 2.8. Aroma Release

**Figure 10:**
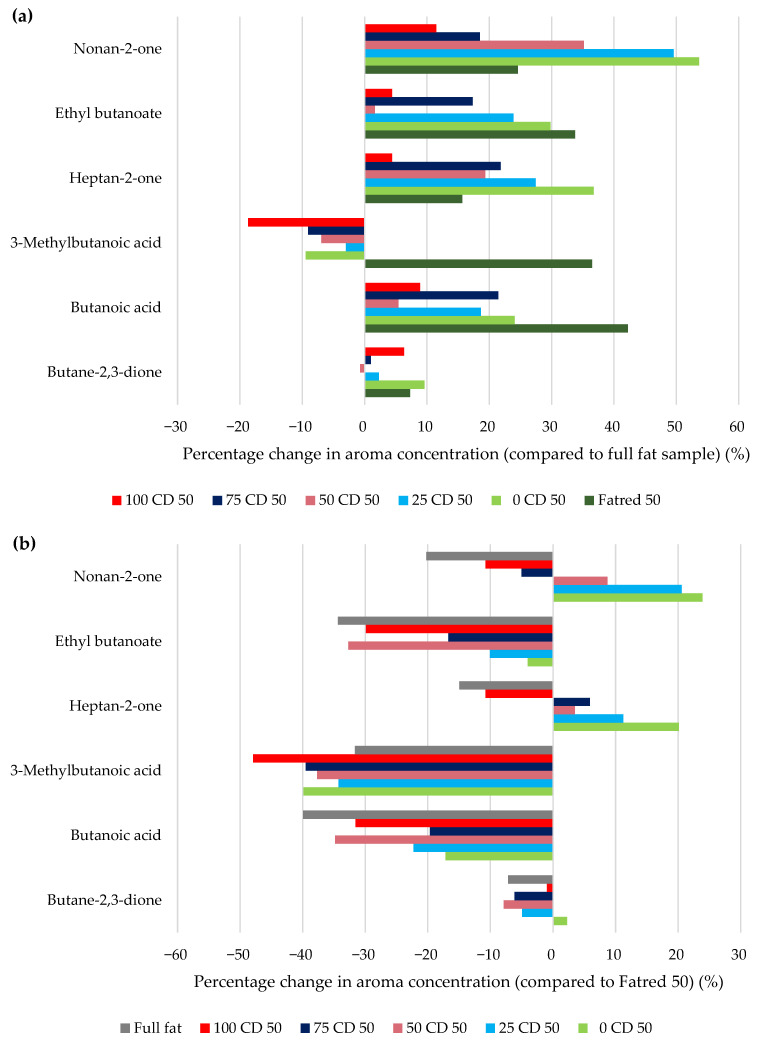
Percentage change in the headspace aroma concentration of the full-fat and 50% reduced-fat samples (Fatred 50 and Y CD 50 samples) compared to the full fat sample (**a**) and the Fatred 50 sample (**b**).

The aroma release of the six cheese aroma compounds was determined by dynamic headspace measurements. The PTR-MS allows us to detect very low concentrations due to its high sensitivity, and enables real-time aroma release measurements from real foods or model systems [53]. The average headspace aroma concentration of every aroma compound of the full-fat sample and the samples with a 50% fat reduction (Fatred X and Y CD 50) can be found in the Appendix A (Table A2). The differences (%) of each of these formulations, compared to the full-fat sample, were used to evaluate the effect of fat reductions and CD concentrations on aroma release, as shown in Figure 10a. Positive deviations from the full-fat spread indicate an enhanced aroma release, whereas negative values demonstrate a reduced release.

Interestingly, only the aroma compounds heptan-2-one and nonan-2-one showed some significant differences. However, trends could be identified for the other compounds. The lower the CD concentration, i.e., the higher the lactose concentration, the higher the aroma release, especially for the more lipophilic aroma compounds, such as heptan-2-one, ethyl butanoate, and nonan-2-one. Piccone et al. [54] also found an increase in the release of four aroma compounds when adding lactose to a solution, explained by a salting-out effect. 3-Methylbutanoic acid was reduced in its release, compared to the full-fat sample, and its retention increased with increasing CD concentrations. The differences in the aroma release are not due to differences in the viscosity, because all Y CD 50 samples exhibited similar viscosity, but can be attributed to different interactions with either CD or lactose. The aroma release from the 100 CD 50 sample was the most similar to the release from the full-fat sample, indicating a similar interaction capacity of CD and fat with the aroma compounds.

Butane-2,3-dione was the least affected by the formulations, whereas the more lipophilic compound nonan-2-one was most affected (Figure 10a). Interestingly, the release of 3-methylbutanoic acid from all Y CD 50 samples was reduced, while the release increased from the Fatred 50 sample. For a different view, Figure 10b shows the percentages of differences in the aroma release of the full-fat and the Y CD 50 samples, compared to the Fatred 50 sample. The release of all aroma compounds was reduced in the full-fat sample, compared to the Fatred 50 sample. An increase in fat is often linked with a decrease in the volatility of hydrophobic compounds [36], whereas in emulsions, the effect of the fat content on hydrophilic aroma compounds is generally small [55]. Since not only the lipophilic compounds were reduced in their release, but also the more hydrophilic ones, the retention of the aroma compounds is probably not only explained by the higher fat content, but also by the significantly higher viscosity of the full-fat sample and, thus, a reduced diffusion of the compounds. 3-Methylbutanoic acid was almost not affected by changes in the ratios of CD and lactose, but was, in general, greatly reduced in its release compared to the Fatred 50 sample. This could be an indication of a lower degree of interaction between 3-methylbutanoic acid and CD or lactose, but the release was more affected by the viscosity of the PCS samples. However, the more lipophilic compounds heptan-2-one and nonan-2-one exhibited an increased aroma release at lower CD concentrations. Conversely, the reduced release at higher CD concentrations indicates an interaction between CD and these two aroma compounds, probably due to hydrophobic effects [56], and a lower impact of the viscosity. Butanoic acid and ethyl butanoate were both reduced in their aroma release, but to a lower degree, with decreasing CD concentrations, indicating an interaction with CD, as well as an influence of viscosity. The results show that the fat content can be reduced by 50% without significantly changing the aroma release. However, some trends were identified. Further studies are necessary to verify that this is a suitable method to analyze the aroma release under oral conditions, with a high correlation to human-sensory data.

### 2.9. Cryo-Scanning Electron Microscope (Cyro-SEM)

**Figure 11:**
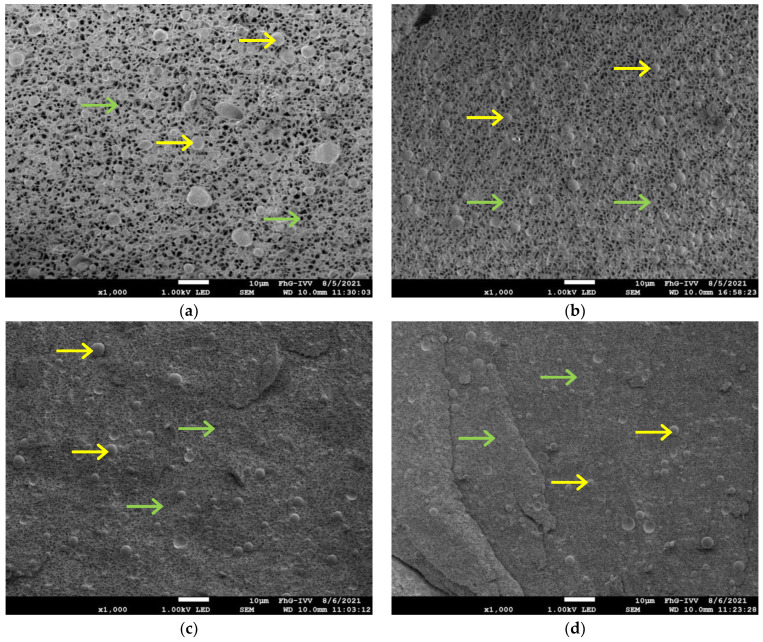
SEM pictures of the full-fat (**a**), 100 CD 50 (**b**), 50 CD 50 (**c**), and 0 CD 50 (**d**) samples. Fat droplets (yellow arrows), continuous network (green arrows). Magnitude 1000×, at 1.0 kV. Bar = 10 µm.

All cryo-SEM images in Figure 11 were taken at 1000× magnification. Figure 11a shows the full-fat sample with fat particles distributed in a continuous protein network. The other three images (Figure 11b–d) show the samples 100 CD 50 (b), 50 CD 50 (c), and 0 CD 50 (d), which are the reduced-fat samples with different ratios of CD and lactose. The sample 100 CD 50 shows an open-pore network similar to the full-fat samples, but with a lower amount of distributed fat particles. The pores in the protein network of the full-fat sample were about 0.37 µm^2^ and 0.33 µm^2^ for the 100 CD 50 sample. Several researchers also found that the protein matrix became denser and more compact as the fat content of cheese decreased, with decreasing spacers that were once occupied by fat globules [40,57,58,59,60]. As the concentration of CD decreased and that of lactose increased, the protein network became denser with more, but smaller, cavities. The pore size for the 50 CD 50 sample was 0.13 µm^2^, and for the 0 CD 50 sample, it was only 0.10 µm^2^. However, a denser protein network was not linked with higher firmness, stickiness, viscosity, gel strength, or aroma release in this study; rather the opposite. Nevertheless, the reduced-fat PCS sample with solely CD as the fat replacer showed a structure more similar to the full-fat PCS, with only slight changes in texture properties and aroma release. It appears that the use of CD in this concentration is most promising for the development of reduced-fat PCS.

## 3. Materials and Methods

### 3.1. Materials

For the production of the model processed cheese spread (PCS) samples, the following ingredients were used: deionized water, vegetable fat (refined, non-hydrogenated) (Akoroma HM, palm oil, 100% fat, from AAK AarhusKarlshamn AB, Malmö, Sweden), rennet casein (90 mesh, minimum protein content of 78% and a maximum content of 12% moisture, 1.5% fat, and 1% lactose from LACTOPROT Deutschland GmbH, Kaltenkirchen, Germany), skim milk powder (protein content of 34%, lactose content of 53% from Andreas Zoellner backstars.de, Bellenberg, Germany), emulsifying salt (JOHA S4, poly- and diphosphate, ICL, BK Giulini GmbH, Ludwigshafen, Germany), table salt (Salta Siede Speisesalz, Suedwestdeutsche Salzwerke AG, Heilbronn, Germany) and lactose (pharmacopoeia quality). The supplier kindly donated the fat replacer corn dextrin (CD) (Nutriose FM 06, partially acidic hydrolyzed corn starch with an average molecular weight of 5 kDa, 82 to 88% of dietary fibers, a content of mono- and disaccharides of 0.3%, and <0.1% starch from Roquette Frères, Lestrem, France).

For the artificial saliva, potassium chloride, potassium dihydrogen phosphate, sodium bicarbonate, magnesium chloride hexahydrate, ammonium carbonate, calcium chloride dihydrate, and hydrochloric acid (Sigma-Aldrich, Munich, Germany) were used.

The aroma compounds butane-2,3-dione (diacetyl), butanoic acid (butyric acid), and ethyl butanoate (ethyl butyrate) were obtained from Fluka (Steinheim, Germany) and 3-methylbutanoic acid (isovaleric acid), heptan-2-one, and nonan-2-one from Sigma-Aldrich (Taufkirchen, Germany). All aroma compounds had a purity of at least 98% or higher.

### 3.2. Preparation of the Model Processed Cheese Spread

All PCS samples were produced in the same manner and the formulations are shown in Table 2. Water and, when indicated, lactose and CD were mixed and heated to 55 °C at a speed level of 1.5 (150 rpm) for 4 min in a Thermomix TM5 blender cooker (Vorwerk Elektrowerke GmbH and Co. KG, Wuppertal, Germany). After the fat addition (as solid fat), the mixture was stirred for another 4 min with the same settings. Then rennet casein was added, and the speed level was increased to 2.5 (350 rpm) for 2 min at 55 °C. Finally, emulsifying salt, skim milk powder, table salt, and aroma solutions were added. The temperature setting was adjusted to 85 °C and the mixture was heated and stirred at a speed level of 6.0 (3100 rpm) for 280 s. The hot mixture was filled in sealable beakers and cooled for 5 min at room temperature, and then stored one week at 1 °C. Subsequently, the samples were stirred gently with a spatula and stored at 5 °C for at least one day before measurements. The cheese spread samples were produced in batch sizes of 500 g and each formulation was produced at least three times. The abbreviations for the samples are listed below. Fatred X denotes all samples where fat is replaced 100% by water, and 100 CD X includes all samples where fat is replaced 100% by CD. X represents the different fat reduction levels. Y CD 50 is used for all samples with a 50% fat replacement, where Y represents the different ratios of CD to lactose.

### 3.3. Preparation of the Aroma Solutions

Two aroma mixtures were prepared to account for the different solubility behaviors of the selected aroma compounds, and to reach the concentrations listed in Table 3, as described in detail by Schädle et al. [22]. Stock solutions were prepared for each aroma compound. Butane-2,3-dione and butanoic acid were dissolved in the buffer solution, whereas the other four compounds were dissolved in ethanol. The stock solutions for solution 1 were diluted with the buffer solution, and for solution 2, with ethanol. An amount of 5 g of the aroma solution 1 and 0.5 g of aroma solution 2 was added to the PCS sample to reach the final concentrations in the 500 g sample, which had been determined in previous studies. Critically, the concentration of ethanol in the emulsion did not exceed 1.6 µL∙g^−1^ and, therefore, did not present a limiting factor for the PTR-MS analyses (see later).

### 3.4. Preparation of Artificial Saliva

Stock solutions and the final artificial saliva were prepared according to the composition and instruction for simulated salivary fluids by Minekus et al. [62]. After preparing the stock solutions, they were mixed to reach the final concentration of the artificial saliva, and the pH was adjusted to a value of pH 7 with hydrochloric acid. The stock solution of calcium chloride dihydrate was prepared separately, because precipitation might occur in the artificial saliva, and it was added later when the artificial saliva was mixed with the sample. The concentrations in the final artificial saliva were 15.1 mmol∙L^−1^ potassium chloride, 3.7 mmol∙L^−1^ potassium dihydrogen phosphate, 13.6 mmol∙L^−1^ sodium bicarbonate, 0.15 mmol∙L^−1^ magnesium chloride hexahydrate, 0.06 mmol∙L^−1^ ammonium carbonate, 1.5 mmol∙L^−1^ calcium chloride dihydrate, and 1.1 mmol∙L^−1^ hydrochloric acid.

### 3.5. Compositional Analysis and pH Measurements

The dry matter content of the PCS samples was determined according to AOAC [63] with a thermo-gravimetrical system at 105 °C (TGA 701, Leco Instrumente GmbH, Mönchengladbach, Germany). The protein content was calculated based on the nitrogen content determined according to the Dumas combustion method, as described by AOAC [64] using a Nitrogen Analyzer TruMac N (Leco Instrumente GmbH, Mönchengladbach, Germany) and a conversion factor of N × 6.25. The fat content was determined based on the method of Caviezel, DGF C-III 19 (00) [65] with slight modifications. In addition to the method of Caviezel, the fats were derivatized with trimethylsulfonium hydroxide before being analyzed by gas chromatography. The pH of the PCS samples was measured using a 206-pH2 digital pH meter (Testo SE and Co. KGaA, Lenzkirch, Germany).

### 3.6. Texture Analysis

**Figure A3:**
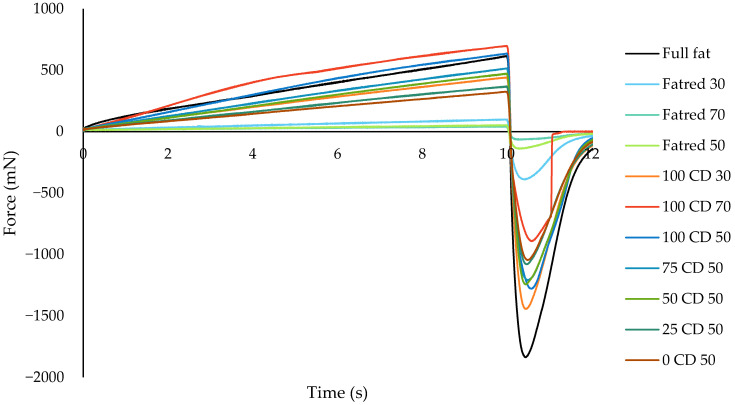
Force–time diagram of single measurements of all PCS formulations as example curves for the texture analysis.

Firmness and stickiness were determined with a Texture Analyzer TA.XTplusC (Stable Micro Systems, Godalming, UK) with a 10 kg load cell at room temperature. A plastic cylinder with a diameter of 12.7 mm (*p*/0.5–½” DIA CYLINDER DELRIN, Stable Micro Systems) was used as measurement probe, applying an instrument protocol for the comparison of the firmness and stickiness of cheese spreads (Exponent connect software version 8.0.3.0, Stable Micro Systems) with slight adjustments. The PCS sample (5 °C) was filled into a tube (inner diameter of 26 mm) and smoothed off at the top. The pre-test speed was set to 2 mm∙s^−1^, the test speed to 1 mm∙s^−1^, and the post-test speed to 10 mm∙s^−1^. The measuring probe entered the sample for a distance of 10 mm and then returned to the starting position. The firmness was determined as the positive peak force of the penetration. The stickiness was the negative peak force at the withdrawal of the probe, known as the resistance force, described with negative values. Example curves of the texture analysis measurements can be found in the Appendix A (Figure A3). Each sample batch was measured at least eight times.

### 3.7. Spreadability

**Figure A4:**
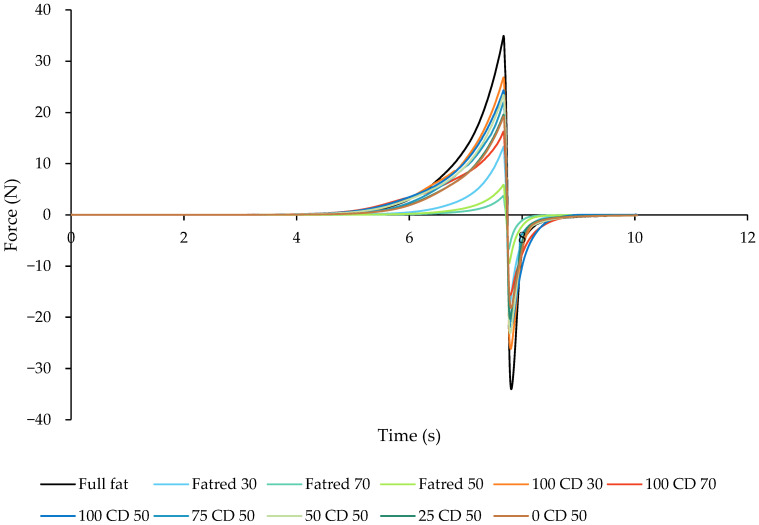
Force–time diagram of single measurements of all PCS formulations as example curves for the spreadability analysis.

Spreadability was determined at room temperature with the same Texture Analyzer TA.XTplusC mentioned above, equipped with the Spreadability Rig (Stable Micro Systems), applying the protocol for the “Spreadability of cheese spread” (Exponent connect software version 8.0.3.0, Stable Micro Systems). A detailed description of the setup and procedure can be found in Schädle et al. [21]. Example curves of the spreadability measurements can be found in the Appendix A (Figure A4). Each sample batch was measured at least eight times.

### 3.8. Flow Behavior

The flow behavior of the PCS samples was determined with a Rapid Visco Analyzer (RVA 4500, Perkin Elmer, Rodgau, Germany). An aliquot of 25 g PCS was weighed into the cups and the following protocol was applied at a temperature of 20 °C: 30 s at 0 rpm, 2 min at 80 rpm. The apparent viscosity was calculated as an average from 90 s to 140 s after the start of the measurement. Each sample batch was measured at least four times.

### 3.9. Dynamic Oscillatory Rheology

**Figure A5:**
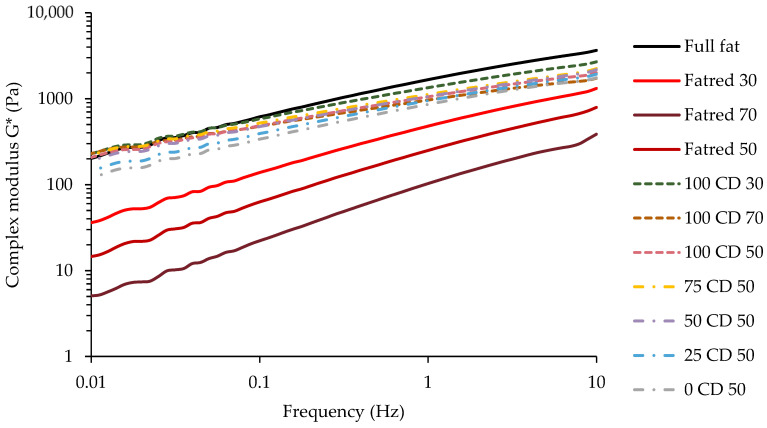
Frequency sweep: Complex modulus G* for single measurements of all PCS formulations as example curves for dynamic oscillatory rheological analysis.

**Figure A6:**
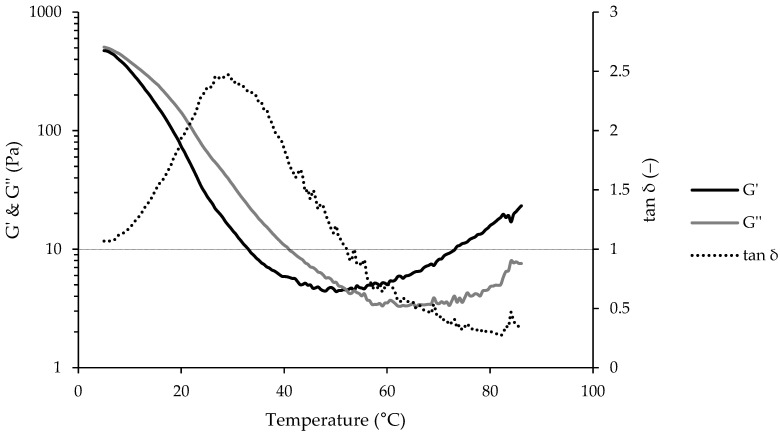
Temperature sweep: Storage modulus G′, loss modulus G″, and loss tangent tan δ of a single measurement of a full fat sample as an example curve for dynamic oscillatory rheological analysis.

The rheological properties of the PCS samples were measured using a Physica MCR 301 rheometer (Anton Paar Germany GmbH, Ostfildern, Germany), equipped with a serrated parallel plate geometry (PP25/P2, d = 25 mm). A Peltier heating system (P-PTD200) and a Peltier hood (H-PTD200) were used for accurate temperature control. The system was held at 5 °C and the samples was applied on the lower plate. After adjusting the gap to 1 mm and trimming the sample, a low-viscosity vegetable oil was applied to the exposed cheese surface to avoid desiccation during measurement. A rest time of 5 min allowed additional loading stress to dissipate. The measurements were conducted within the linear viscoelastic region, as determined by an amplitude sweep (with a constant frequency at 0.1 Hz and a varying deformation of 0.01 to 100%). To perform a frequency sweep, the frequency was changed logarithmically from 10 to 0.01 Hz, with a slope of 20 points per decimal power, at a constant amplitude of 0.1% and a temperature of 5 °C. After a rest time of 30 s at a frequency of 0.1 Hz and an amplitude of 0.1%, a temperature sweep was performed at the same frequency and amplitude settings. The temperature was increased linearly from 5 to 86 °C with a heating rate of 3 °C∙min^−1^. At least three measurements were carried out for each sample batch. Example curves of the frequency sweep (Figure A5) and the temperature sweep (Figure A6) can be found in the Appendix A. The storage modulus (G′), loss modulus (G″), and loss tangent (tan δ) were determined. The complex modulus (G*) was calculated using the formula (Equation (1)):

(1) $$G^{*}=\sqrt{{\left(G^{\prime }\right)}^{2}+{\left(G^{\prime \prime }\right)}^{2}},$$

To evaluate the changes in the viscoelastic properties of the PCS samples, Winter’s critical gel theory (Equation (2)) was used, where the complex modulus can be expressed as:

(2) $$G^{*}\left(\omega \right)=A_{F}\;\cdot \;\omega^{\frac{1}{z}},$$

where A_F_ is the strength of the gel (Pa∙s^1/z^), ω is the angular frequency (s^−1^) (ω=2πf, with an oscillation frequency f (Hz)) and z is the interaction factor (–), which is defined as the number of structure units interacting with one another in a three-dimensional network. The higher the interaction factor, the more interactions occur in the matrix of the PCS sample [11,44,66,67]. For the estimations of A_F_ and z, a non-linear regression analysis was performed.

### 3.10. Tribological Properties

The tribological properties of the PCS samples were measured, as described by Schädle et al. [21]. An aliquot of 1 g of model PCS was used and, in deviation from the described method, the normal force was set to 7 N. The normal force in our setup is divided over three pins with an angle of 45°, resulting in a force perpendicular to the pin surface of about 1.65 N for each pin. The coefficient of friction (COF) was determined as a function of the sliding speed, and three curves were determined for each aliquot (referred to as curve 1, 2, and 3). Each sample batch was measured four times and with a new set of three PDMS pins for each batch.

### 3.11. Aroma Release Analysis by Proton-Transfer-Reaction Mass Spectrometry (PTR-MS)

The release dynamics of the six target aroma compounds were studied using a high sensitivity proton-transfer-reaction mass spectrometer (hs-292 PTR-MS; IONICON Analytik GmbH, Innsbruck, Austria). The analyses were carried out following the procedure reported by Schädle et al. [22] with the following adaptions. The PCS sample was mixed with artificial saliva in a ratio of 50:50 (*w*/*v*), as Minekus et al. [62] suggested for food in the oral phase: An aliquot of 25 g PCS was mixed with 20 mL of artificial saliva, 125 µL calcium chloride dihydrate stock solution, and 4.875 mL water, and was homogenized for 1.5 min at 15,000 rpm with an Ultra-Turrax (T25 digital, IKA-Werke GmbH & Co. KG, Staufen, Germany). Each sample batch was measured three times.

### 3.12. Cryo-Scanning Electron Microscope (Cyro-SEM)

An aliquot of about 5 mg PCS sample was filled in a small aluminum beaker, and air bubbles were removed with a needle. The beaker was subsequently fixed at the cryo shuttle and was plunged into slush liquid nitrogen at atmospheric pressure (−196 °C) and further cooled down to −210 °C. The frozen specimen was transferred into the cryo preparation unit (PP2000, Quorum Technologies Ltd., Lewes, UK) and freeze-fractured within a high vacuum (10^−3^ to 10^−4^ Pa) at a temperature of −160 to −140 °C with a cold scalpel blade or a manipulator. The surface water of the fractured specimen was sublimated at a temperature of −80 °C. The sublimation time varied between the samples in the range of 30 to 60 min, and the surface structure was regularly observed and checked in the SEM. Subsequently, the specimen was sputter coated with 5 nm platinum and transferred into the SEM (JSM 7200F, Jeol GmbH, Freising, Germany). The cryo stage was cooled at a temperature range of −160 to −140 °C and the accelerating voltage was 1 kV. The pore size of the protein network was analyzed with the ImageJ image analysis software, version 1.53 n [68], according to an adapted procedure of El-Bakry et al. [69]. The scale of the measurements was set based on the scale bars on the cryo-SEM images. These images were converted into binary images and their thresholds were adjusted to show the protein network. The area of the pores in the network was analyzed to calculate the average pore size [69,70].

### 3.13. Statistical Analysis

The Shapiro–Wilk test was used to confirm the Gaussian distribution, and the Levene test was applied to test for the homogeneity of variance. The data were subsequently processed by a single factor (univariate) analysis of variance (ANOVA) followed by Tukey’s honest significance test (α = 0.05). The data are expressed as the mean ± standard deviation. The Pearson correlation coefficient between the results was determined to evaluate the possible correlations.

## 4. Conclusions

We have investigated the rheological, textural, and tribological properties, as well as the spreadability, flow behavior, and aroma release in a model processed cheese spread (PCS) with three fat reduction levels, where fat was replaced by either water, corn dextrin, or various ratios of corn dextrin and lactose. The results showed that the addition of CD and lactose changed the viscosity, gel, and lubrication properties, as well as the aroma release. In particular, high CD concentrations had an impact on firmness, stickiness, gel strength, interaction factor, flowability (with heating), melting temperature, and the aroma release of hydrophobic aroma compounds. The sole use of CD was determined to be promising for fat reduction in PCS products, as it adequately mimics the properties of fat. Replacing 30% or 50% fat with CD did not change the firmness, nor the aroma release of the PCS, but the sample became easier to spread and less sticky. However, the viscosity of the full-fat PCS could not be achieved with CD, and an additional thickener would be required to obtain a similar thickness. The observed correlations can contribute to reducing the analytical effort and streamlining the developing process. In addition, the findings on CD as a fat replacer have the potential to facilitate the development of appealing reduced-fat processed cheese products and, likely, other dairy-based emulsion products.

Further studies on the aroma release and sensory perceptions are necessary to verify the results under in vivo conditions. The present study suggests a complex interaction between aroma compounds, PCS ingredients, and textural properties. Therefore, for the development of new reduced-fat PCS, those characteristics should be taken into account to predict the impact on overall sensory characteristics.

## Figures and Tables

**Table 1:** Values of gel strength A_F_ and the interaction factor z of Winter’s critical gel theory, as well as the values of storage modulus G′, loss modulus G″, complex modulus G*, and loss tangent tan δ at the reference frequency of 1 Hz of the PCS samples.

Formulation	A_F_	z	G′ at 1 Hz	G″ at 1 Hz	G* at 1 Hz	tan δ at 1 Hz
	(Pa∙s^1/z^)	(–)	(Pa)	(Pa)	(Pa)	(–)
Full fat	859.5 ± 179.6 ^a^	2.66 ± 0.08 ^g^	1450.7 ± 298.3 ^a^	1040.9 ± 170.1 ^a^	1785.9 ± 340.9 ^a^	0.72 ± 0.04 ^d^
Fatred 30	206.6 ± 43.0 ^e^	2.18 ± 0.07 ^h^	350.8 ± 72.9 ^e^	352.4 ± 59.9 ^d^	497.4 ± 93.8 ^d^	1.01 ± 0.04 ^c^
Fatred 70	36.3 ± 6.2 ^f^	1.77 ± 0.10 ^j^	57.7 ± 8.9 ^f^	89.2 ± 10.4 ^f^	106.2 ± 13.5 ^e^	1.55 ± 0.06 ^a^
Fatred 50	93.7 ± 17.6 ^f^	1.97 ± 0.08 ^i^	156.0 ± 34.8 ^f^	192.4 ± 29.6 ^e^	247.9 ± 44.9 ^e^	1.25 ± 0.08 ^b^
100 CD 30	649.5 ± 56.0 ^b^	2.99 ± 0.06 ^de^	1063.1 ± 86.9 ^b^	648.8 ± 37.8 ^b^	1245.5 ± 93.7 ^b^	0.61 ± 0.02 ^efg^
100 CD 70	643.6 ± 73.9 ^bc^	3.64 ± 0.09 ^a^	983.9 ± 104.9 ^bc^	461.9 ± 31.6 ^c^	1087.1 ± 108.4 ^bc^	0.47 ± 0.02 ^i^
100 CD 50	606.1 ± 57.7 ^bc^	3.31 ± 0.07 ^b^	955.7 ± 88.5 ^bcd^	508.1 ± 39.2 ^c^	1082.4 ± 96.0 ^bc^	0.53 ± 0.01 ^h^
75 CD 50	560.8 ± 43.2 ^bcd^	3.16 ± 0.06 ^c^	901.9 ± 65.9 ^bcd^	508.5 ± 24.7 ^c^	1035.5 ± 69.4 ^c^	0.56 ± 0.01 ^gh^
50 CD 50	538.3 ± 52.9 ^cd^	3.07 ± 0.07 ^cd^	875.2 ± 80.9 ^cd^	515.3 ± 33.7 ^c^	1015.7 ± 86.7 ^c^	0.59 ± 0.02 ^fg^
25 CD 50	480.9 ± 36.5 ^d^	2.93 ± 0.08 ^ef^	792.8 ± 53.6 ^d^	503.8 ± 25.0 ^c^	939.4 ± 58.5 ^c^	0.64 ± 0.01 ^ef^
0 CD 50	483.6 ± 30.0 ^d^	2.87 ± 0.05 ^f^	802.0 ± 47.9 ^d^	527.6 ± 24.2 ^c^	960.0 ± 53.2 ^c^	0.66 ± 0.01 ^e^

The data are expressed as mean ± standard deviation (*n* = 9). Values followed by different letters in a column indicate significant differences between samples (*p* ≤ 0.05) following one-way ANOVA (Tukey).

**Table 2:** Formulations of the processed cheese spread (PCS) samples (CD = corn dextrin).

							Y CD 50				
		Fatred X			100 CD X						
	Full Fat	Fatred 30	Fatred 70	Fatred 50	100 CD 30	100 CD 70	100 CD 50	75 CD 50	50 CD 50	25 CD 50	0 CD 50
	(g/100 g)	(g/100 g)	(g/100 g)	(g/100 g)	(g/100 g)	(g/100 g)	(g/100 g)	(g/100 g)	(g/100 g)	(g/100 g)	(g/100 g)
Water	61.4	67.4	75.4	71.4	61.4	61.4	61.4	61.4	61.4	61.4	61.4
Fat	20	14	6	10	14	6	10	10	10	10	10
CD	0	0	0	0	6	14	10	7.5	5	2.5	0
Lactose	0	0	0	0	0	0	0	2.5	5	7.5	10
Rennet casein	9.5	9.5	9.5	9.5	9.5	9.5	9.5	9.5	9.5	9.5	9.5
Skim milk powder	3.5	3.5	3.5	3.5	3.5	3.5	3.5	3.5	3.5	3.5	3.5
Emulsifying salt	2.5	2.5	2.5	2.5	2.5	2.5	2.5	2.5	2.5	2.5	2.5
Table salt	2	2	2	2	2	2	2	2	2	2	2
Aroma solution 1	1	1	1	1	1	1	1	1	1	1	1
Aroma solution 2	0.1	0.1	0.1	0.1	0.1	0.1	0.1	0.1	0.1	0.1	0.1

**Table 3:** Concentrations of aroma compounds in the aroma solutions 1 and 2, the resulting final concentrations in the model PCS samples and the log *P* value of each compound.

Aroma Solution	Aroma Compound	Concentration in Aroma Solution (µg∙g^−1^)	Concentration in PCS (µg∙g^−1^)	Log *P* Value [61]
1	Butane-2,3-dione	1938	19.4	−1.34
	Butanoic acid	8437	84.4	0.79
	3-Methylbutanoic acid	28,867	288.7	1.16
	Ethyl butanoate	1500	15.0	1.71
2	Heptan-2-one	5245	5.3	1.98
	Nonan-2-one	56,404	56.4	3.16

## Data Availability

The data presented in this study is available on request from the corresponding author.

## Abbreviations

CD	Corn dextrin
Full fat	100% fat sample
Fatred X	All samples where fat is replaced 100% by water
Fatred 30	30% fat is replaced 100% by water
Fatred 70	70% fat is replaced 100% by water
Fatred 50	50% fat is replaced 100% by water
100 CD X	All samples where fat is replaced 100% by CD
100 CD 30	30% fat is replaced 100% by CD
100 CD 70	70% fat is replaced 100% by CD
100 CD 50	50% fat is replaced 100% by CD
75 CD 50	50% fat is replaced 75% by CD and 25% by lactose
50 CD 50	50% fat is replaced 50% by CD and 50% by lactose
25 CD 50	50% fat is replaced 25% by CD and 75% by lactose
0 CD 50	50% fat is replaced 0% by CD and 100% by lactose
Y CD 50	All samples with 50% fat replacement
PCS	Processed cheese spread

## Appendix A

**Table A1:** Composition of the samples based on 100 g model processed cheese spread (abs = absolute, DM = dry matter, CD = corn dextrin).

Formulation	DM	Fat (abs)	Fat (in DM)	Protein (abs)	Protein (in DM)	Energy Density ^1^	Energy Density ^1^
Per 100 g	(g)	(g)	(g)	(g)	(g)	(kJ)	(kcal)
Full fat	37.0 ± 1.5 ^a^	20.9 ± 0.5 ^a^	56.5 ± 2.6 ^a^	8.9 ± 0.1 ^a^	24.0 ± 0.8 ^d^	942	228
Fatred 30	30.4 ± 0.8 ^b^	14.8 ± 0.3 ^b^	48.7 ± 1.7 ^b^	8.7 ± 0.3 ^a^	28.5 ± 0.7 ^bc^	720	174
Fatred 70	23.9 ± 3.1 ^c^	6.5 ± 0.3 ^d^	27.4 ± 3.4 ^d^	8.9 ± 0.1 ^a^	37.5 ± 4.0 ^a^	424	102
Fatred 50	26.8 ± 1.0 ^bc^	9.8 ± 0.5 ^c^	36.8 ± 3.2 ^c^	8.8 ± 0.1 ^a^	32.8 ± 1.0 ^b^	572	138
100 CD 30	36.3 ± 1.3 ^a^	14.5 ± 0.3 ^b^	40.0 ± 2.0 ^c^	8.9 ± 0.1 ^a^	24.5 ± 1.0 ^cd^	770	186
100 CD 70	37.2 ± 1.4 ^a^	6.4 ± 0.0 ^d^	17.6 ± 0.3 ^e^	8.9 ± 0.4 ^a^	24.0 ± 1.5 ^cd^	541	130
100 CD 50	37.1 ± 1.7 ^a^	10.2 ± 0.2 ^c^	27.5 ± 1.1 ^d^	8.5 ± 0.2 ^a^	23.0 ± 1.7 ^d^	656	158
75 CD 50	35.9 ± 0.8 ^a^	10.4 ± 0.2 ^c^	28.9 ± 0.4 ^d^	8.8 ± 0.1 ^a^	24.4 ± 0.6 ^cd^	675	163
50 CD 50	36.6 ± 0.8 ^a^	10.5 ± 0.3 ^c^	28.8 ± 0.8 ^d^	8.9 ± 0.1 ^a^	24.3 ± 0.9 ^cd^	694	167
25 CD 50	35.9 ± 1.4 ^a^	10.1 ± 0.1 ^c^	28.1 ± 1.4 ^d^	8.9 ± 0.1 ^a^	24.8 ± 1.2 ^cd^	714	172
0 CD 50	36.6 ± 2.2 ^a^	10.3 ± 0.4 ^c^	28.3 ± 2.7 ^d^	8.9 ± 0.2 ^a^	24.4 ± 1.0 ^cd^	733	176

^1^ Energy density was calculated using the manufacturer specifications of the ingredients and the respective formulations of the samples.

The data are expressed as mean ± standard deviation (*n* = 9). Values followed by different letters in a column indicate significant differences between samples (*p* ≤ 0.05) following one-way ANOVA (Tukey).

**Table A2:** Concentrations of the six target aroma compounds in the headspace gas of the full-fat sample and the samples with 50% fat reduction.

Formulation	Butane-2,3-dione (ppb_v_)	Butanoic Acid (ppb_v_)	3-Methylbutanoic Acid (ppb_v_)	Heptan-2-one (ppb_v_)	Ethyl Butanoate (ppb_v_)	Nonan-2-one (ppb_v_)
Full fat	114.0 ± 5.0 ^a^	93.8 ± 6.2 ^a^	37.7 ± 19.8 ^a^	82.6 ± 13.0 ^c^	130.4 ± 19.0 ^a^	119.9 ± 28.7 ^b^
100 CD 50	121.3 ± 16.6 ^a^	102.1 ± 34.2 ^a^	29.5 ± 5.5 ^a^	85.9 ± 9.8 ^bc^	136.3 ± 38.6 ^a^	131.3 ± 34.5 ^ab^
75 CD 50	115.2 ± 7.2 ^a^	113.8 ± 20.2 ^a^	32.1 ± 7.8 ^a^	99.1 ± 14.7 ^abc^	153.6 ± 29.0 ^a^	138.3 ± 29.0 ^ab^
50 CD 50	113.1 ± 7.1 ^a^	93.0 ± 28.9 ^a^	32.7 ± 4.0 ^a^	97.3 ± 10.4 ^abc^	132.6 ± 38.9 ^a^	155.0 ± 22.0 ^ab^
25 CD 50	116.6 ± 10.8 ^a^	111.2 ± 20.7 ^a^	33.8 ± 7.8 ^a^	103.4 ± 17.1 ^ab^	162.3 ± 44.3 ^a^	169.3 ± 37.2 ^a^
0 CD 50	125.1 ± 10.9 ^a^	116.3 ± 35.7 ^a^	32.0 ± 7.2 ^a^	110.4 ± 16.0 ^a^	170.3 ± 56.7 ^a^	173.4 ± 33.6 ^a^
Fatred 50	122.4 ± 11.4 ^a^	133.2 ± 5.0 ^a^	44.8 ± 12.8 ^a^	94.5 ± 6.8 ^abc^	175.6 ± 15.2 ^a^	144.4 ± 12.9 ^ab^

The data are expressed as mean ± standard deviation (*n* = 9). Values followed by different letters in a column indicate significant differences between samples (*p* ≤ 0.05) following one-way ANOVA (Tukey).
